# Supplementary material for: Single-photon emission from two-dimensional perovskites channeled through low-energy edge states
Source: Nat Commun. 2026 Mar 23;17:4317. doi: 10.1038/s41467-026-71000-2 (PMC13172483; doi:10.1038/s41467-026-71000-2)
Supplement: Supplementary file 1 — Supplementary Information [file 41467_2026_71000_MOESM1_ESM.pdf]

Supplementary Information

Single-photon emission from two-dimensional perovskites channeled through low-energy edge states

Gunwoo Na[1†], Jee Yung Park[2,3,4†], Jae-Pil So[5], Peijun Guo[3,4], Letian Dou[2,6,7*], Hong-Gyu Park[1*]

[1]Department of Physics and Astronomy, and Institute of Applied Physics, Seoul National University, Seoul 08826, Republic of Korea.

[2]Davidson School of Chemical Engineering, Purdue University, West Lafayette, IN 47907, United States.

[3]Department of Chemical and Environmental Engineering, Yale University, New Haven, CT 06511, United States.

[4]Energy Sciences Institute, Yale University, West Haven, CT 06516, United States.

[5]Department of Physics, Soongsil University, Seoul 06978, Republic of Korea.

[6]Department of Chemistry, Purdue University, West Lafayette, IN 47907, United States.

[7]Department of Chemistry, Emory University, Atlanta, GA 30322, United States.

*Corresponding authors. Email: letian.dou@emory.edu (L.D.); hgpark@snu.ac.kr (H.-G.P.)

†These authors contributed equally to this work.

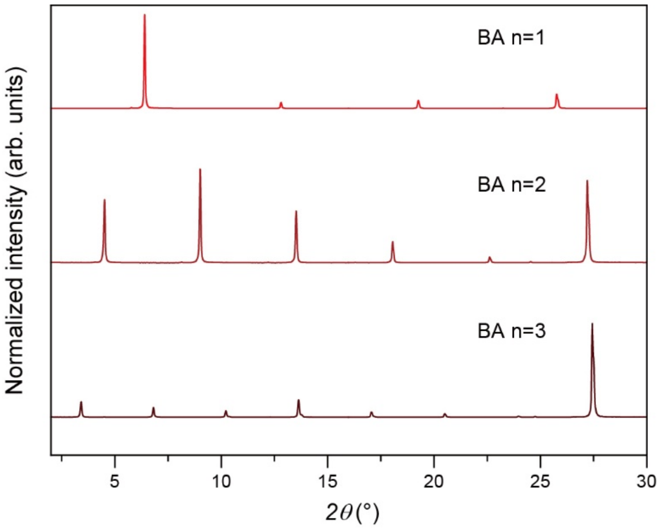

Supplementary Fig. 1. X-ray diffraction patterns of single-crystal sheets with BA *n*=1, 2, and 3. Powder X-ray diffraction (PXRD) patterns of phase-pure bulk single crystals synthesized via a slow cooling crystallization method. The results confirm the high compositional purity of each single-crystal batch, which were vacuum-dried and mechanically exfoliated into thin sheets for measurements. Each plot shows the 2θ range from 2° to 30°.

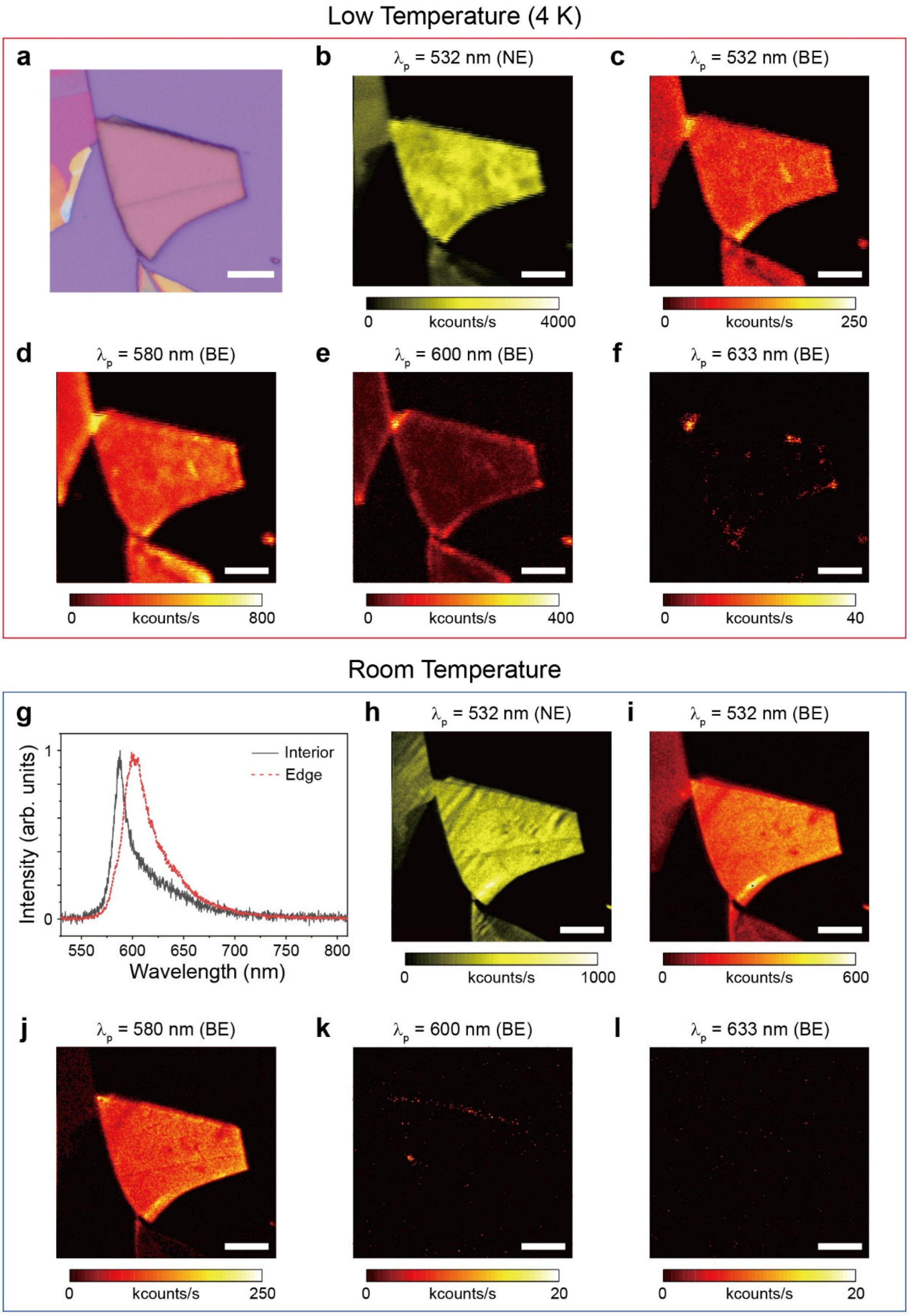

Supplementary Fig. 2. Temperature-dependent spatial PL mapping of BA *n*=2 at different pump wavelengths. a, Optical microscope image of an exfoliated BA *n*=2 single-crystal flake used for PL mapping. Scale bar, 10 μm. **b-f,** Low-temperature (4 K) spatial PL intensity maps.

Narrow emission (NE) map under pulsed excitation at λ_p = 532 nm acquired with a 600 nm short-pass filter (**b**). Broad emission (BE) maps under excitation at λ_p = 532 nm (**c**), 580 nm (**d**), 600 nm (**e**), and 633 nm (**f**), acquired with a 650 nm long-pass filter. Scale bars, 10 μm. **g-l,** Room-temperature measurements on the same flake. PL spectra acquired from the interior (solid black) and edge (dashed red) regions under excitation at λ_p = 532 nm (**g**). NE map at λ_p = 532 nm with a 600 nm short-pass filter (**h**). BE maps at λ_p = 532 nm (**i**), 580 nm (**j**), 600 nm (**k**), and 633 nm (**l**), acquired with a 650 nm long-pass filter. Scale bars, 10 μm.

These results suggest that low temperature does not fundamentally change the main features of the edge states. Under various pump wavelengths (532, 580, 600, and 633 nm), broad emission from the flake edges is also enhanced at room temperature, similar to the low-temperature case. Localized emission spots are observed at the edges even at room temperature, although their brightness under 600 nm and 633 nm excitation is weaker than at 4 K. These observations indicate that the edge-state-related emission mechanism is already present at room temperature.

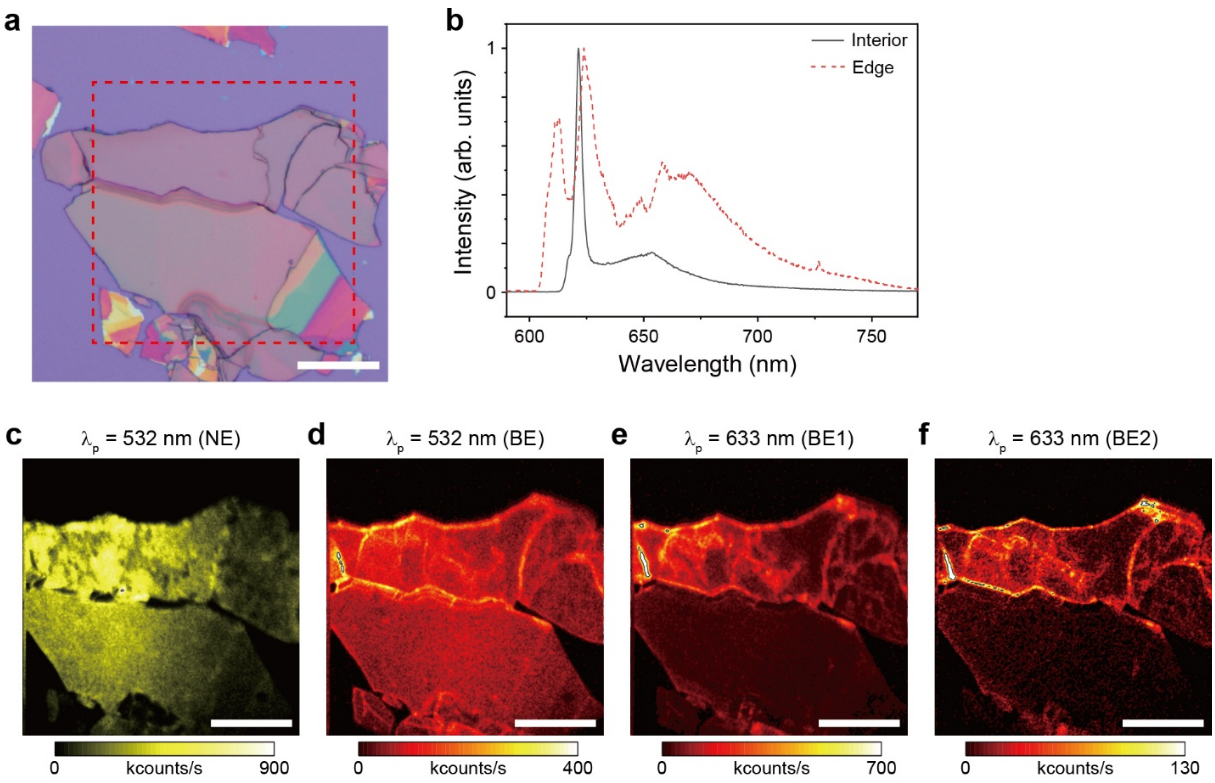

Supplementary Fig. 3. Spatial mapping of PL intensity from BA *n*=3 at various pump wavelengths. a, Optical microscope image of an exfoliated single crystal used for measurements. Scale bar, 50 μm. **b,** PL spectra exhibiting narrow emission from the interior (solid black) and broad emission from the edge (dotted red). **c,** Spatial PL map of the narrow emission component (NE) at the pump wavelength (λ_p) of 532 nm, using a 630 nm band-pass filter. Scale bar, 50 μm. **d-f,** Spatial PL maps of the broad emission component (BE) at the pump wavelength (λ_p) of 532 nm (**d**), 633 nm (**e**), and 633 nm (**f**), using a 650 nm long-pass filter (**d** and **e**) and a 700 nm long-pass filter (**f**). Scale bar, 50 μm. All measurements are conducted at 4 K.

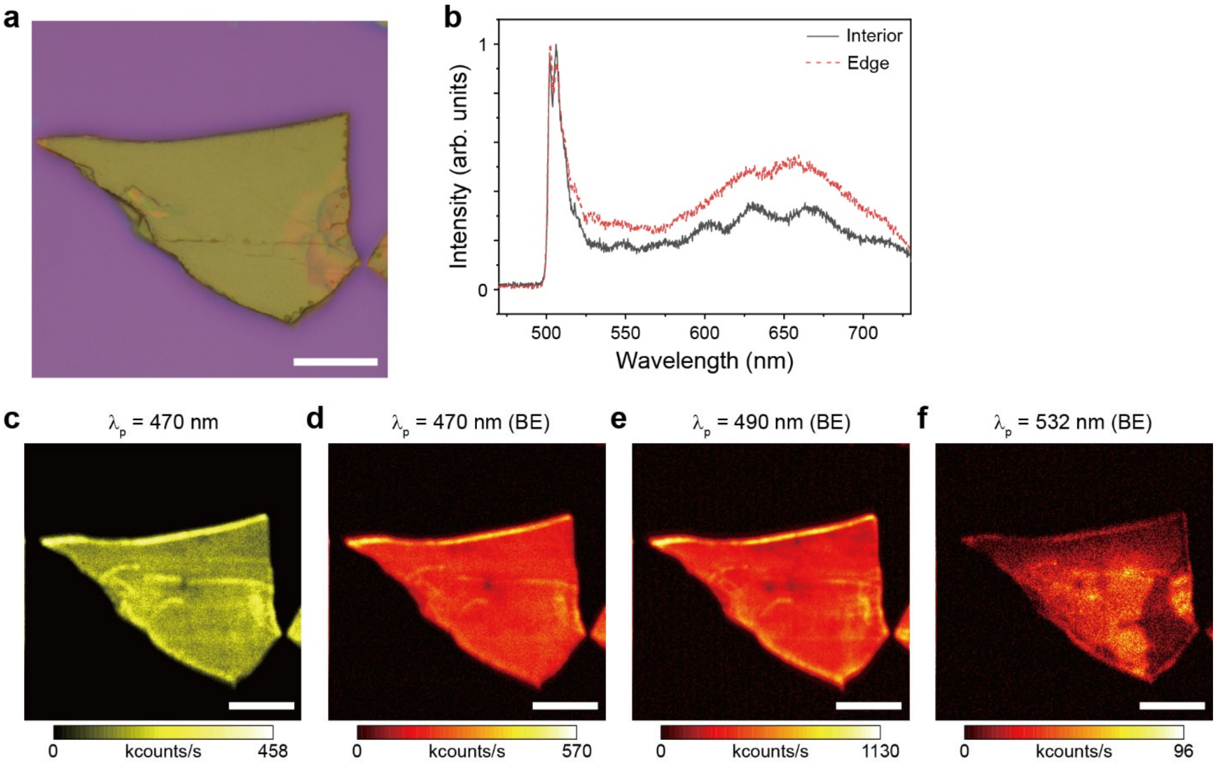

Supplementary Fig. 4. Spatial mapping of PL intensity from BA *n*=1 at various pump wavelengths. a, Optical microscope image of an exfoliated single crystal used for measurements. Scale bar, 20 μm. **b,** PL spectra of the interior (solid black) and the edge (dotted red). No substantial difference was observed between them. **c,** Spatial PL map of the emission component at the pump wavelength (λ_p) of 470 nm, using a 500 nm long-pass filter. Scale bar, 20 μm. **d-f,** Spatial PL maps of the broad emission component (BE) at the pump wavelength (λ_p) of 470 nm (**d**), 490 nm (**e**), and 532 nm (**f**), using a 550 nm long-pass filter. Scale bar, 20 μm. In contrast to $n \geq 2$ perovskites, sub-bandgap excitation did not lead to strong broad emission or spatially confined emission characteristics at the edges. All measurements are conducted at 4 K.

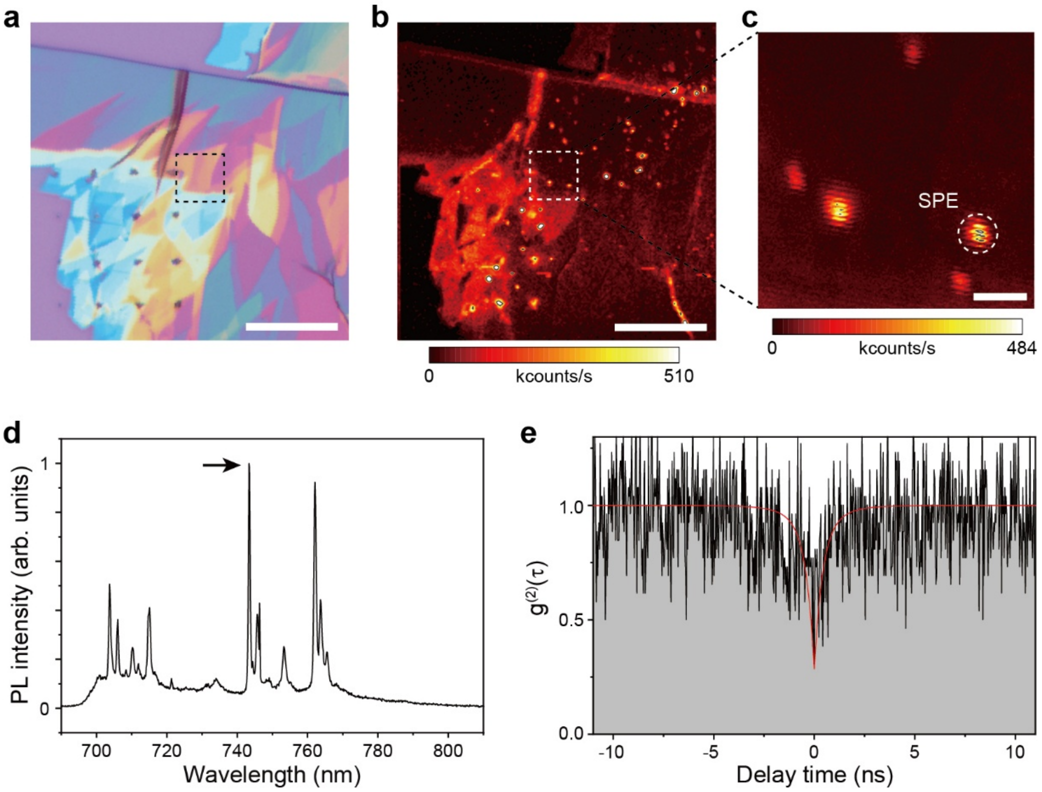

Supplementary Fig. 5. Single-photon emitter located at the edge in BA *n*=3. a, Optical microscope image of an exfoliated BA *n*=3 single crystal on a SiO_2/Si substrate. Scale bar, 10 µm. **b,** Spatial PL map of the black dashed area in (**a**). PL intensity is measured at 4 K at a pump wavelength of 633 nm with a 700 nm long-pass filter. Scale bar, 10 µm. **c,** Magnified view of the white dashed box in (**b**). Scale bar, 1 µm. **d,** PL spectrum for the bright spot in (**c**) (white dashed circle). **e,** Measured second-order correlation function $g^{(2)}(\tau)$ for the sharp peak at 743.4 nm in (**d**) (black arrow). The value of $g^{(2)}(0)$ is 0.278 ± 0.103, indicating the single-photon emission feature.

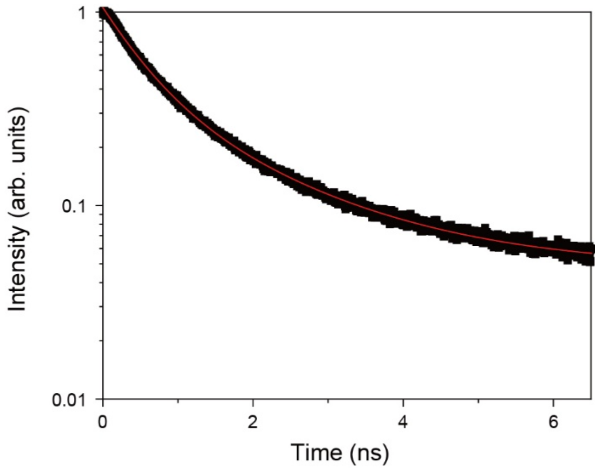

Supplementary Fig. 6. TRPL measurement of a BA *n*=3 emitter. At 4 K, the PL lifetime of BA *n*=3 was measured to be $\tau = 1.729 \pm 0.032$ ns, with the red curve showing an exponential fit. This lifetime is longer than that of BA *n*=2 (~0.87 ns), as shown in Fig. 3f.

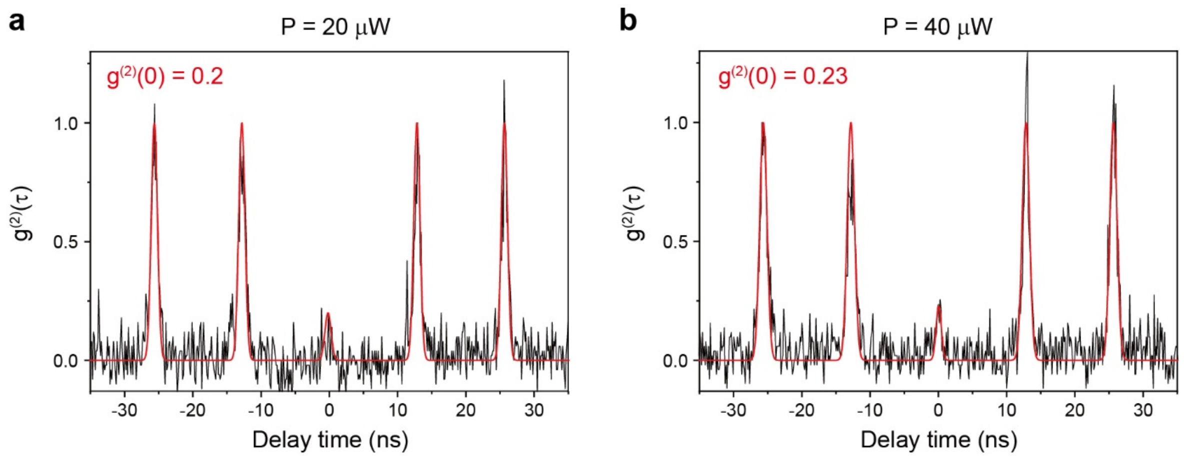

Supplementary Fig. 7. Power-dependent $g^{(2)}(\tau)$ measurements. a-b, Second-order correlation functions $g^{(2)}(\tau)$ were measured at 4 K from a localized emitter (spot 1 in Fig. 4c) under sub-bandgap pulsed excitation at powers of P = 20 μW (**a**) and P = 40 μW (**b**). The $g^{(2)}(0)$ values are 0.20 (**a**) and 0.23 (**b**), indicating improved single-photon purity at lower pump power.

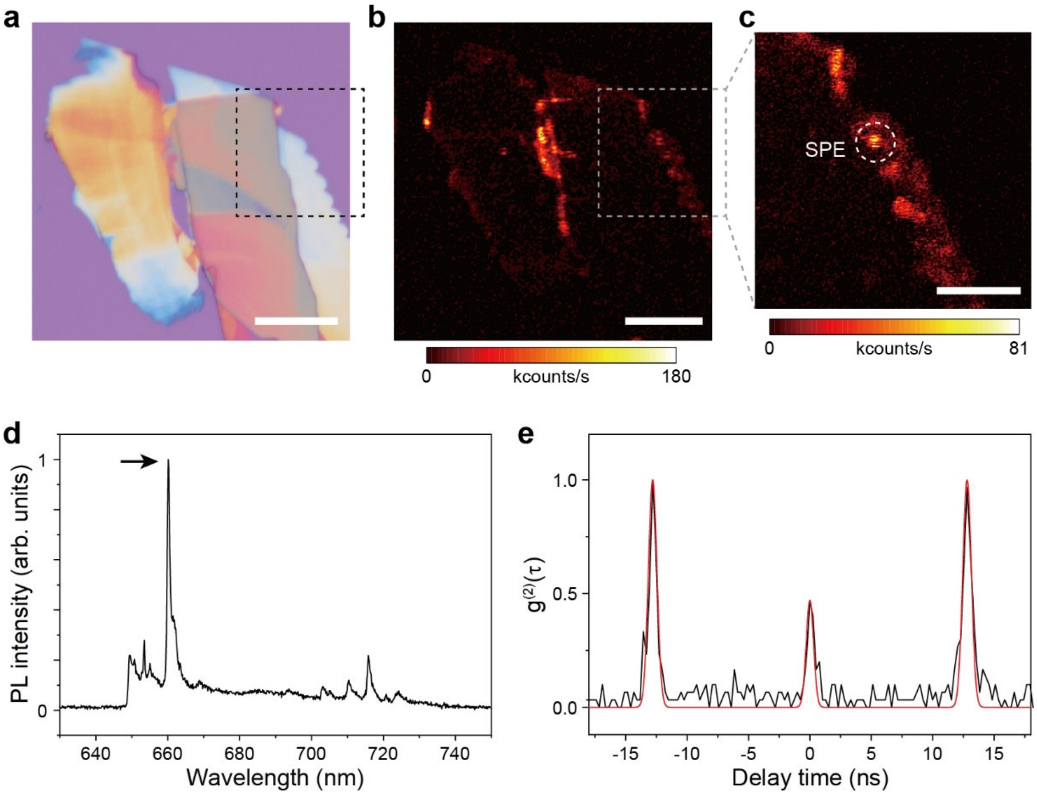

Supplementary Fig. 8. Interior point-defect single-photon emission in BA *n*=2. a, Optical microscope image of an exfoliated BA *n*=2 single crystal on a SiO_2/Si substrate. Scale bar, 10 µm. **b,** Spatial PL map of the black dashed area in (**a**). PL intensity is measured at 4 K at a pump wavelength of 633 nm with a 650 nm long-pass filter. Scale bar, 10 µm. **c,** Magnified view of the gray dashed box in (**b**). Scale bar, 5 µm. **d,** PL spectrum for the bright spot in (**c**) (white dashed circle). **e,** Measured second-order correlation function $g^{(2)}(\tau)$ for the sharp peak at 660.2 nm in (**d**) (black arrow). The value of $g^{(2)}(0)$ is 0.471 ± 0.047.

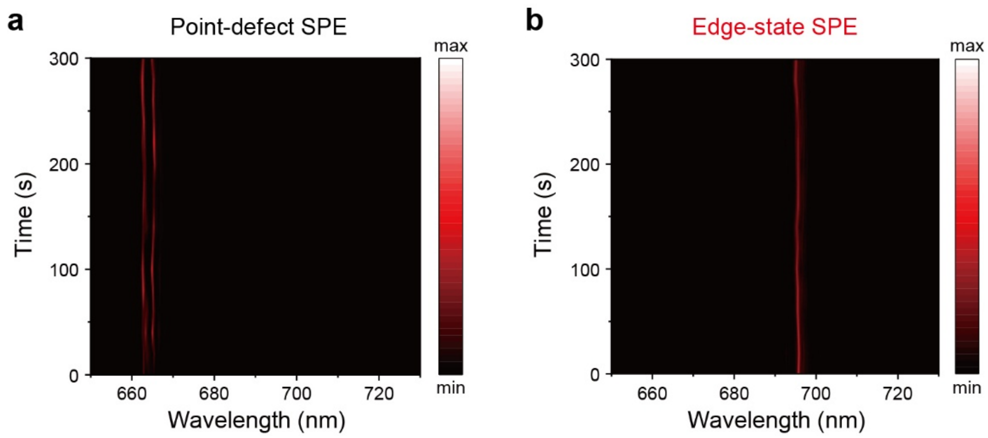

Supplementary Fig. 9. Time-resolved spectral tracking of single-photon emitters. a-b, PL spectra of a point-defect single-photon emitter (**a**) and an edge-state single-photon emitter (**b**) were measured as a function of time for 5 min, with spectra acquired every 10 s. These results indicate that the edge-state single-photon emitter is less prone to bleaching and exhibits improved spectral stability compared with the point-defect single-photon emitter.

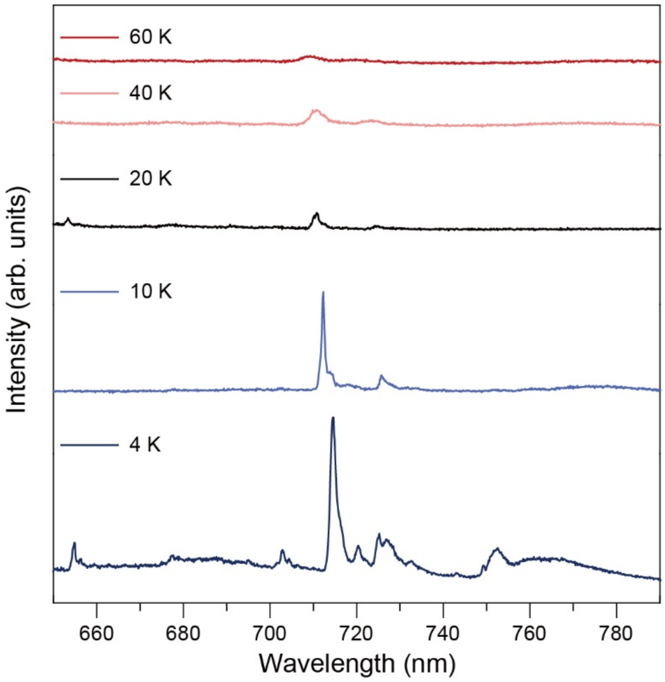

Supplementary Fig. 10. Temperature-dependent PL measurement at the edge with mid-gap states in BA *n*=2. The temperature dependency of the PL spectra, ranging from 4 K to 60 K, was investigated using a pump wavelength of 633 nm and a 650 nm long-pass filter. The excitation powers were 1 μW. The peak at ~714.6 nm disappears above 60 K.

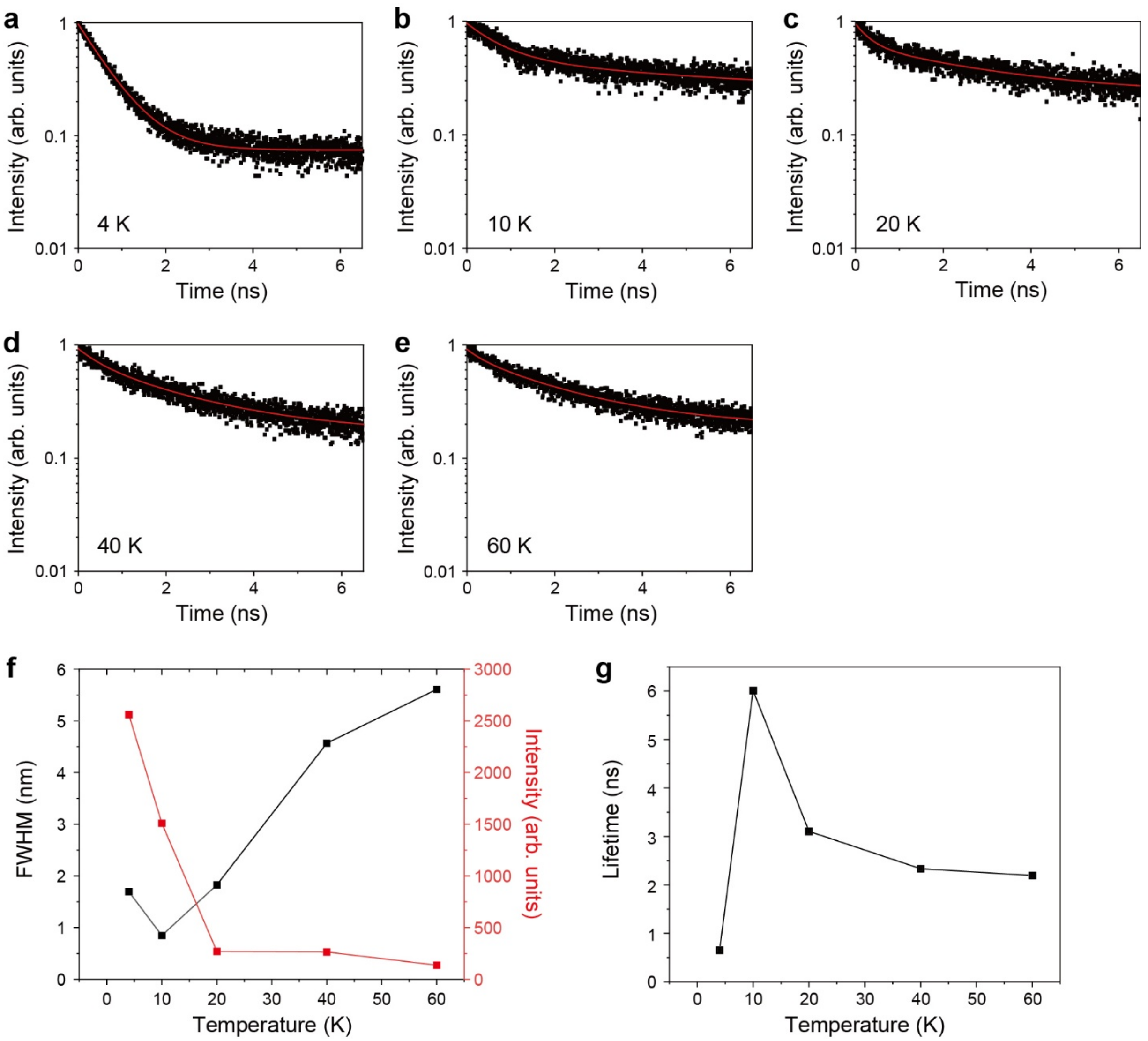

Supplementary Fig. 11. Temperature-dependent TRPL measurements and spectral characteristics. a-e, TRPL measurements on an edge-state emitter in BA n=2 at 4 (**a**), 10 (**b**), 20 (**c**), 40 (**d**), and 60 K (**e**). **f,** Measured emission linewidth (FWHM) (left y-axis, black) and integrated PL intensity (right y-axis, red) as a function of temperature. **g,** Temperature dependence of the PL lifetime extracted from **a-e**.

The results reveal several key features. First, the linewidth (FWHM) increases from 0.8 nm at 10 K to 5.5 nm at 60 K, consistent with enhanced exciton-phonon interactions and increased spectral fluctuations at elevated temperatures. Second, the integrated PL intensity decreases rapidly above 20 K, and the lifetime decreases from ~6 ns near 10 K to ~2 ns at 60 K, indicating that nonradiative pathways become increasingly competitive as temperature rises. Third, the non-monotonic lifetime

trend, an increase from 4 K to 10 K followed by a decrease, suggests a crossover in the dominant decay pathways. Overall, these results indicate that the practical temperature limit is primarily set by phonon-driven loss of spectral selectivity together with activated competition from nonradiative/escape channels, rather than a single mechanism alone.

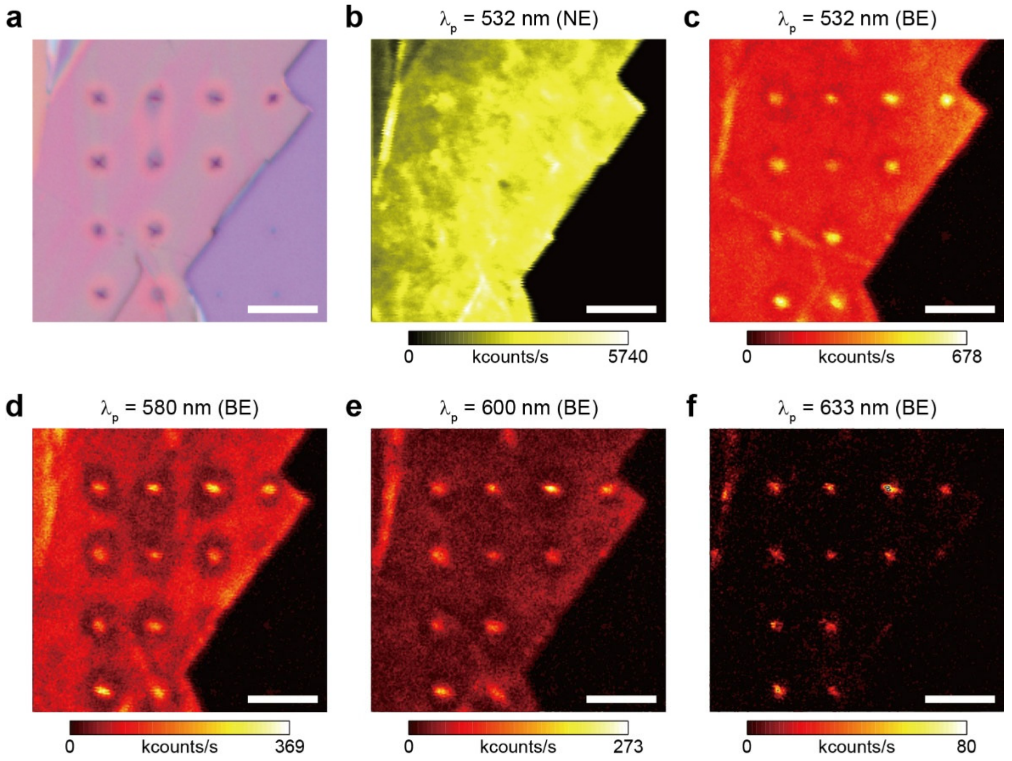

Supplementary Fig. 12. Spatial PL mapping of artificially engineered edges in BA *n*=2 at various pump wavelengths. a, Optical microscope image of a BA *n*=2 single-crystal flake with artificially engineered edges used for PL mapping. Scale bar, 20 μm. **b,** Spatial PL map of the narrow emission component (NE) measured at 4 K under excitation at λ_p = 532 nm using a 600 nm short-pass filter. Scale bar, 20 μm. **c-f,** Spatial PL maps of the broad emission component (BE) measured at 4 K using a 650 nm long-pass filter under excitation at λ_p = 532 nm (**c**), 580 nm (**d**), 600 nm (**e**), and 633 nm (**f**). Scale bar, 20 μm.

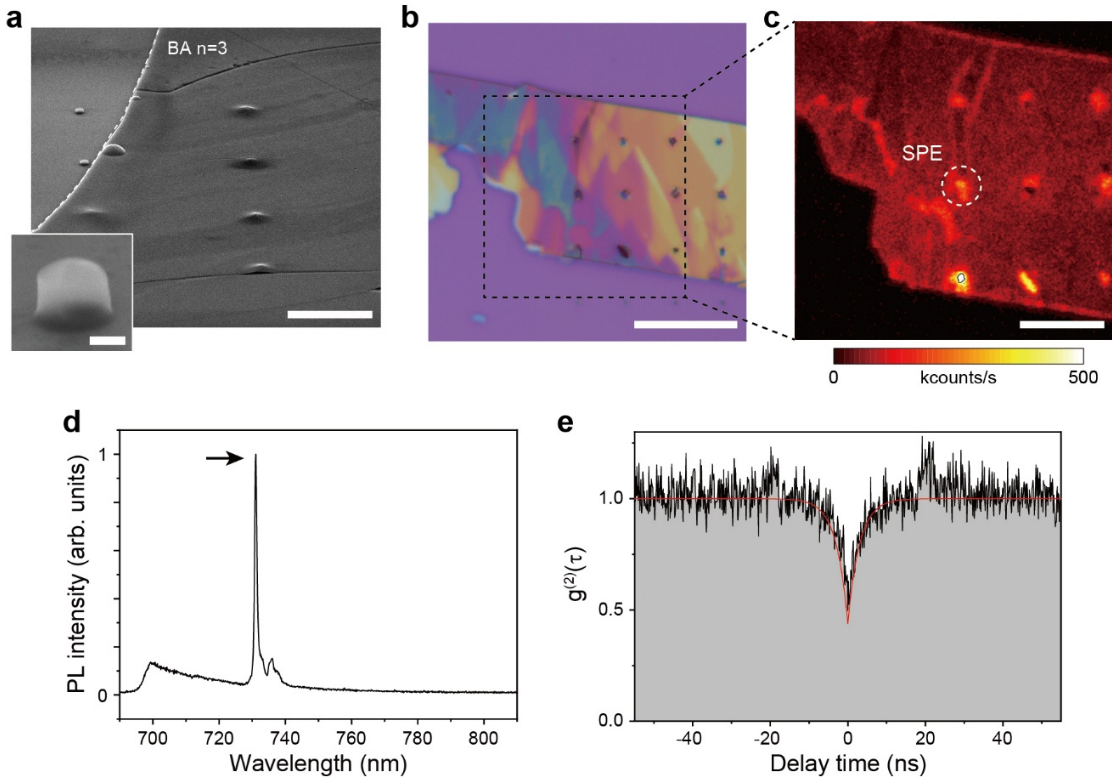

Supplementary Fig. 13. Single-photon emission from BA *n*=3 with artificially engineered edges. a, SEM image of an exfoliated BA *n*=3 single-crystal sheet placed onto a SiO_2/Si substrate with a PMMA nanorod array. The inset shows a magnified image of the fabricated nanorod. Scale bars, 5 μm and 200 nm (inset). **b,** Optical microscope image of the sample. Scale bar, 20 μm. **c,** Spatial PL map of the black dashed area in (**b**). PL intensity is measured at 4 K using a pump wavelength of 633 nm in conjunction with a 700 nm long-pass filter. Scale bar, 10 μm. **d,** PL spectrum measured from the bright emission spot in (**c**) (white dashed circle). A single peak is observed at 731.1 nm. **e,** Measured second-order correlation function $g^{(2)}(\tau)$ from the peak indicated by the black arrow in (**d**). The value of $g^{(2)}(0)$ is 0.438 ± 0.032.

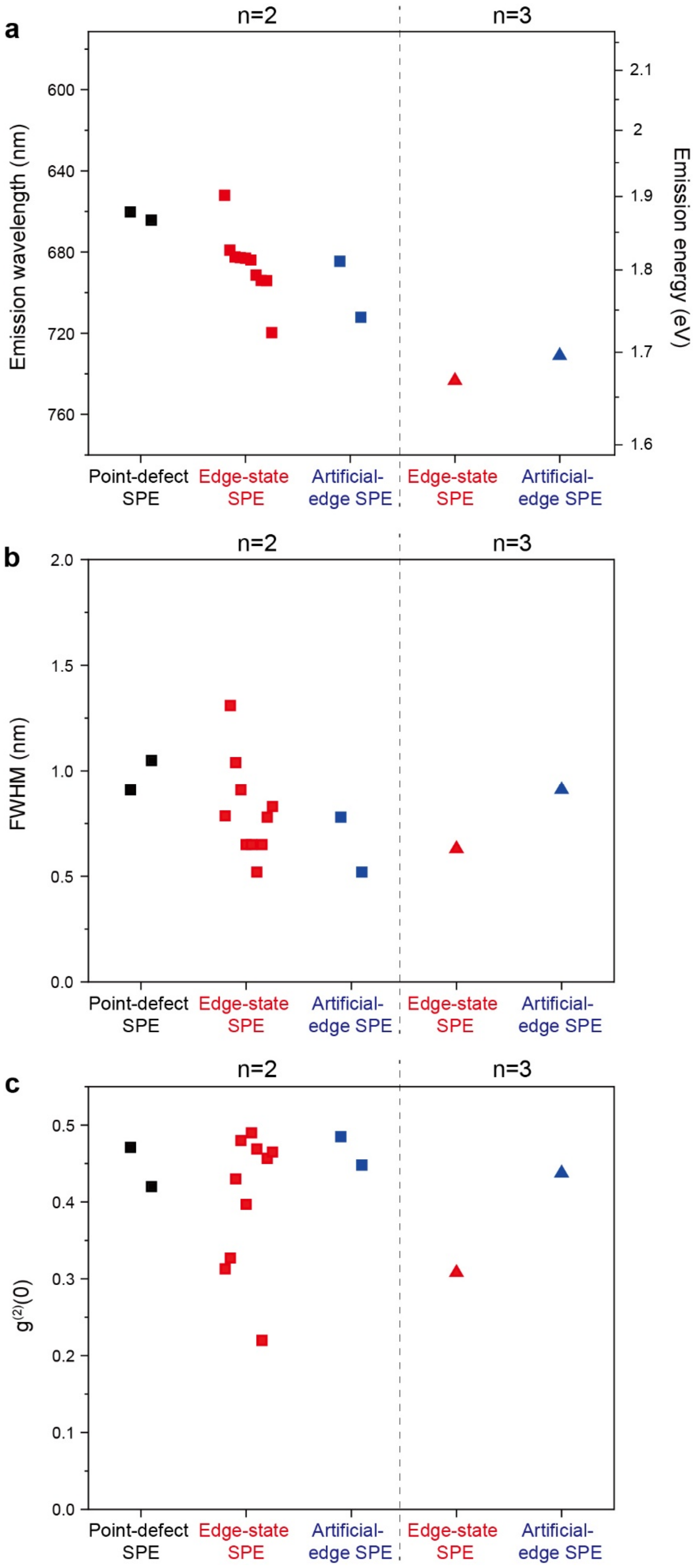
a
n=2
n=3
Emission wavelength (nm)
600
640
680
720
760
Emission energy (eV)
2.1
2
1.9
1.8
1.7
1.6
Point-defect SPE
Edge-state SPE
Artificial-edge SPE
Edge-state SPE
Artificial-edge SPE
b
n=2
n=3
FWHM (nm)
2.0
1.5
1.0
0.5
0.0
Point-defect SPE
Edge-state SPE
Artificial-edge SPE
Edge-state SPE
Artificial-edge SPE
c
n=2
n=3
g(2)(0)
0.5
0.4
0.3
0.2
0.1
0.0
Point-defect SPE
Edge-state SPE
Artificial-edge SPE
Edge-state SPE
Artificial-edge SPE

Supplementary Fig. 14. Statistics of single-photon emitters in 2D perovskites. a-c, Properties of three types of single-photon emitters—interior point-defect, natural edge-state, and artificial edge-state single-photon emitters (SPEs)—summarized across two 2D perovskite compositions (BA n=2 and BA n=3). Shown are (**a**) emission wavelength (left axis) and corresponding emission energy (right axis), (**b**) linewidths (FWHM), and (**c**) $g^{(2)}(0)$ values for the same set of emitters. The dashed line separates the BA n=2 and BA n=3 datasets.

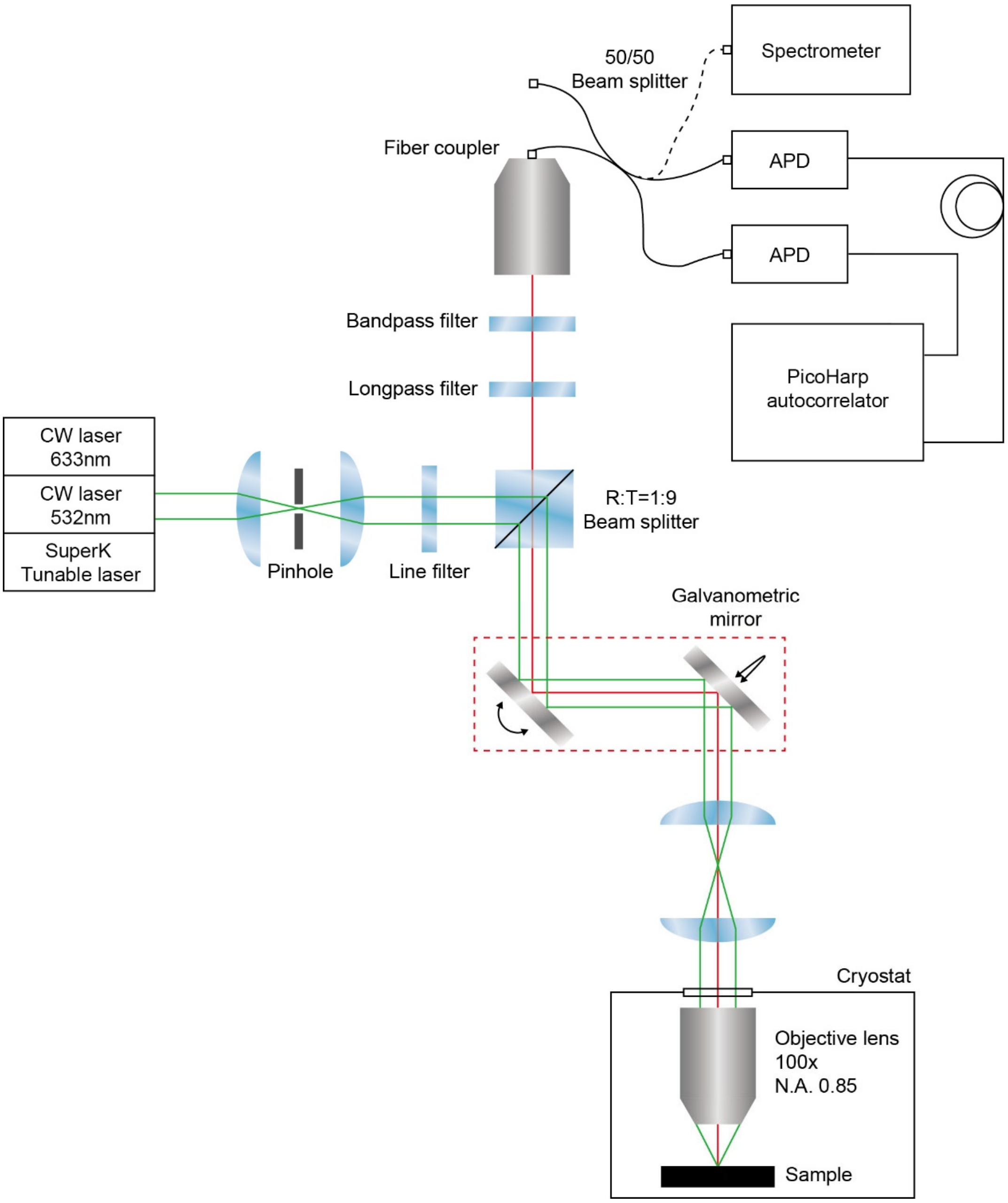

Supplementary Fig. 15. Optical measurement setup. The pump beam (green line) from a 532 nm or 633 nm CW laser or a supercontinuum pulsed laser was focused on the sample with a 100× objective (NA = 0.85) and scanned by a galvanometric mirror. The light emitted from the sample (red line) mounted on a piezoelectric stage in a cryostat was collected by the same objective and directed to a fiber coupler for photon counting, spectral analysis, and photon correlation measurements. Long-pass and band-pass filters removed reflected pump light, achieving a spectral resolution of 0.09 nm.
